# Author Correction: Boosting mechanical durability under high humidity by bioinspired multisite polymer for high-efficiency flexible perovskite solar cells

**DOI:** 10.1038/s41467-026-72048-w

**Published:** 2026-04-13

**Authors:** Zhihao Li, Chunmei Jia, Zhi Wan, Junchao Cao, Jishan Shi, Jiayi Xue, Xirui Liu, Hongzhuo Wu, Chuanxiao Xiao, Can Li, Meng Li, Chao Zhang, Zhen Li

**Affiliations:** 1 State Key Laboratory of Solidification Processing, Northwestern Polytechnical University, Xi’an, China; 2 School of Materials Science and Engineering, Northwestern Polytechnical University, Xi’an, China; 3 School of Civil Aviation, Northwestern Polytechnical University, Xi’an, Shaanxi China; 4 Key Laboratory for Special Functional Materials of Ministry of Education, School of Nanoscience and Materials Engineering, Henan University, Kaifeng, China; 5 Ningbo Institute of Materials Technology and Engineering, Chinese Academy of Sciences, Ningbo, Zhejiang Province China; 6 Ningbo New Materials Testing and Evaluation Center CO., Ltd, Ningbo, Zhejiang Province China

**Keywords:** Solar cells, Electronic devices

Correction to: *Nature Communications* 10.1038/s41467-025-57102-3, published online 19 February 2025

In the version of the article initially published, in the third paragraph of the Introduction, “the hydroscopic perovskite materials” should have read “the hygroscopic perovskite materials”. This correction has now been made to the HTML and PDF versions of the article.

