# Supplementary material for: Boosting mechanical durability under high humidity by bioinspired multisite polymer for high-efficiency flexible perovskite solar cells
Source: Nat Commun. 2025 Feb 19;16:1771. doi: 10.1038/s41467-025-57102-3 (PMC11840045; doi:10.1038/s41467-025-57102-3)
Supplement: Supplementary file 1 — Supplementary Information [file 41467_2025_57102_MOESM1_ESM.pdf]

SUPPLEMENTARY INFORMATION

Boosting Mechanical Durability under High Humidity by Bioinspired Multisite Polymer for High-Efficiency Flexible Perovskite Solar Cells

Zhihao Li[1, 2, 3, 4], Chunmei Jia[1, 2], Zhi Wan[1, 2], Junchao Cao[3], Jishan Shi[1, 2], Jiayi Xue[1, 2], Xirui Liu[5], Hongzhuo Wu[4], Chuanxiao Xiao[5, 6], Can Li[1, 2], Meng Li[4*], Chao Zhang[3*], Zhen Li[1, 2*]

1. State Key Laboratory of Solidification Processing, Northwestern Polytechnical University, Xi'an, 710072, P. R. China
2. School of Materials Science and Engineering, Northwestern Polytechnical University, Xi'an, 710072, P. R. China
3. School of Civil Aviation, Northwestern Polytechnical University, Xi'an, Shaanxi 710072, China
4. Key Laboratory for Special Functional Materials of Ministry of Education, School of Nanoscience and Materials Engineering, Henan University, Kaifeng, 475004, China
5. Ningbo Institute of Materials Technology and Engineering, Chinese Academy of Sciences, Ningbo City, Zhejiang Province, 315201, China
6. Ningbo New Materials Testing and Evaluation Center CO., Ltd, Ningbo City, Zhejiang Province, 315201, China

E-mail: mengli@henu.edu.cn, chaozhang@nwpu.edu.cn, lizhen@nwpu.edu.cn

This PDF file includes:

Supplementary Figures 1 to 45

Supplementary Table 1 to 7

Supplementary Note 1 to 2

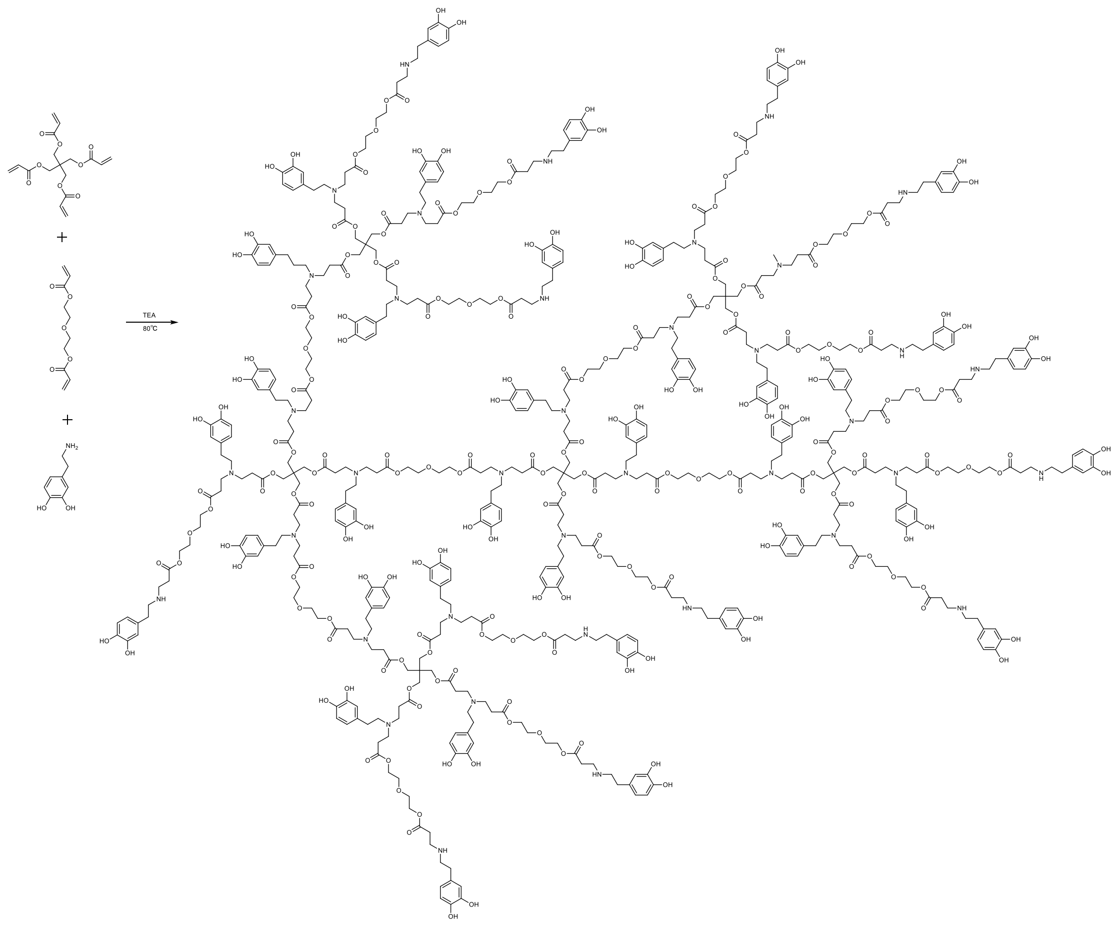

Supplementary Fig. 1. Synthetic scheme of hyperbranched polymer dopamine adhesive (HPDA).

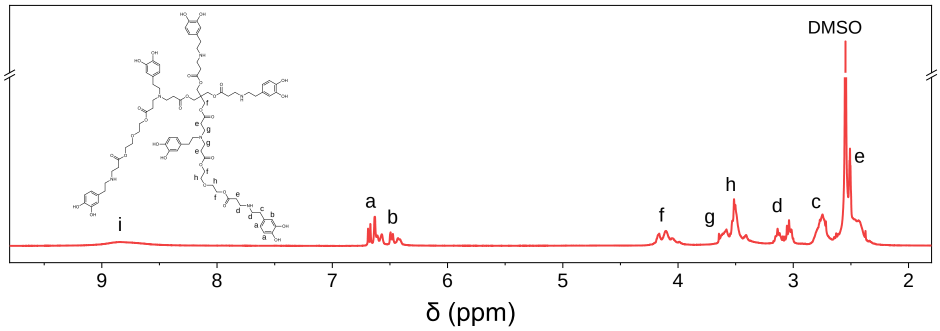

Supplementary Fig. 2. ^{1}H-NMR spectroscopy of the HPDA. The double bond peaks of vinyl group disappear, and the proton peaks in the benzene ring of the catechol group at 6.30–6.62 ppm are observed, hinting that catechol groups have been successfully introduced into the hyperbranched polymer and end-capped the double bonds at the terminal of the HPDA completely.

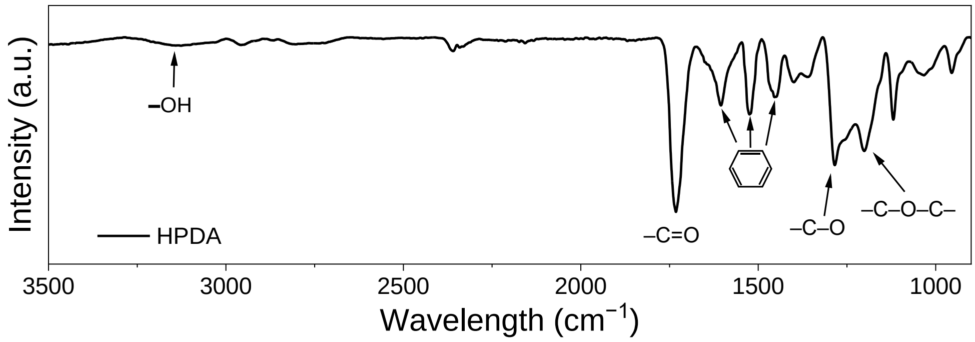

Supplementary Fig. 3. Fourier transform infrared (FTIR) spectrum of the HPDA.

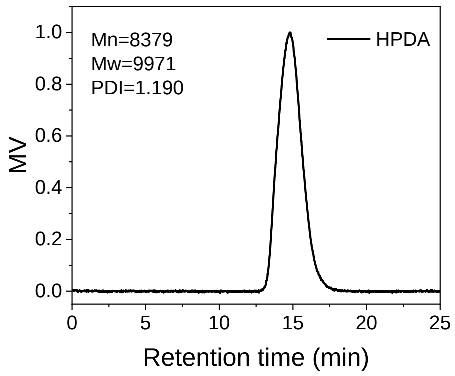

Supplementary Fig. 4. Gel permeation chromatography (GPC) curves of HPDA for characterization of the Molecular weight of HPDA.

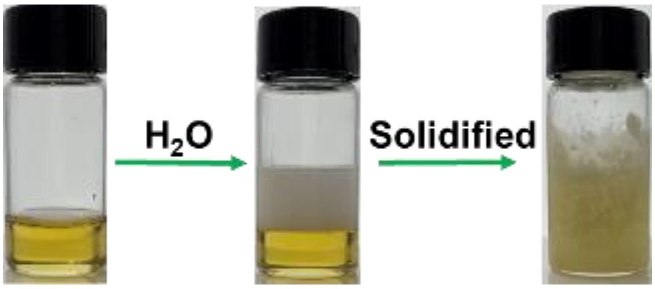

Supplementary Fig. 5. Digital images showing the adhesion of water-triggered coacervation of HPDA adhesives.

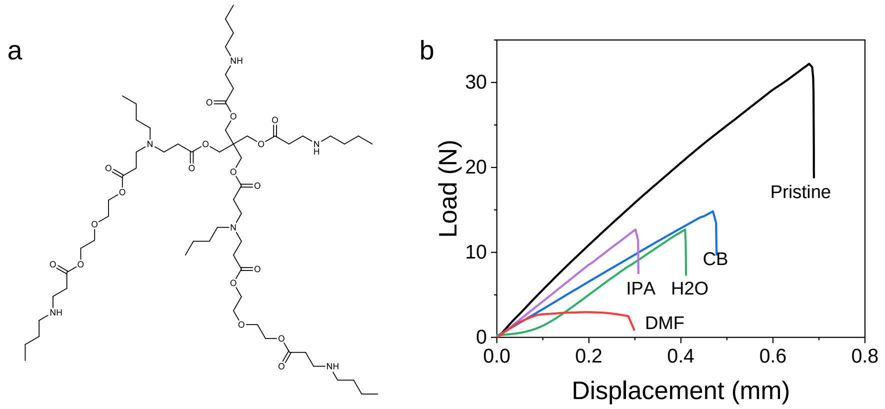

Supplementary Fig. 6. Mechanical characterization of hyperbranched polymer butylamine adhesive (HPBA). (a) Molecular structure of HPBA. (b) Lap shear curve of HPBA bonding to ITO substrates.

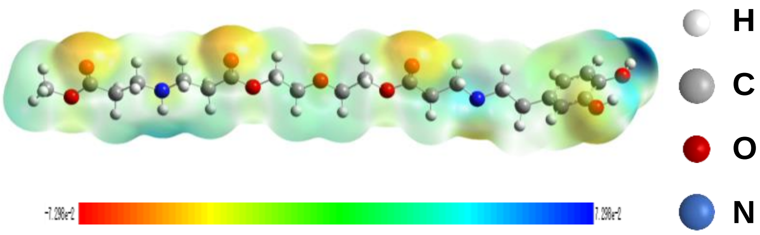

Supplementary Fig. 7. Theoretical calculation of HPDA. Electrostatic potential of the smallest unit of HPDA calculated using the DFT.

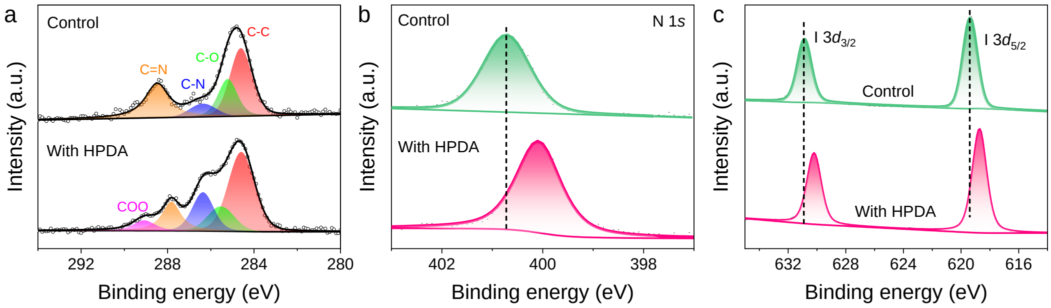

Supplementary Fig. 8. XPS spectra of control and HPDA–modified perovskite films. (a) C 1*s*, (b) N 1*s* and (c) I 3*d*.

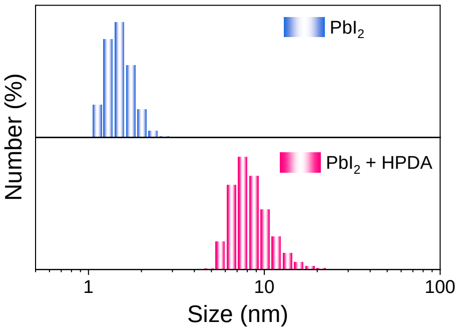

Supplementary Fig. 9. Colloidal size distribution of PbI_2 in DMF/DMSO with and without HPDA.

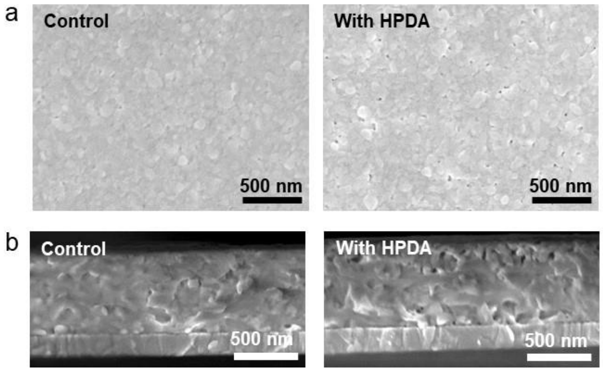

Supplementary Fig. 10. SEM images of PbI_2 film with and without HPDA (a) top–view and (b) cross–sectional.

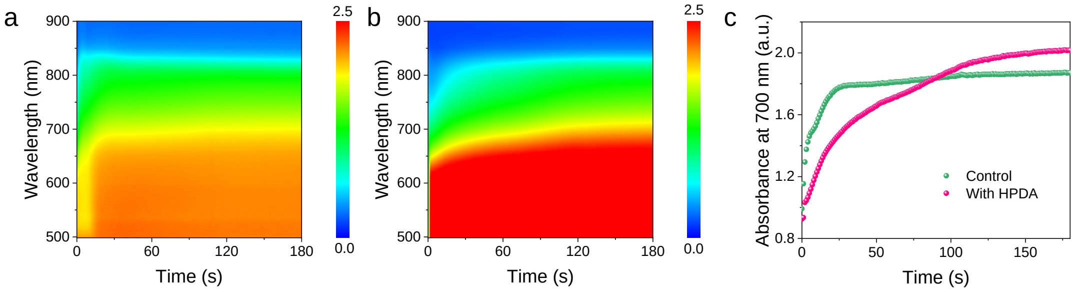

Supplementary Fig. 11. In situ UV-Vis absorption spectra of the control and HPDA-modified perovskite films during the thermal annealing. (a) spectra of control and (b) HPDA-modified perovskite films, (c) The absorbance changes at 700 nm.

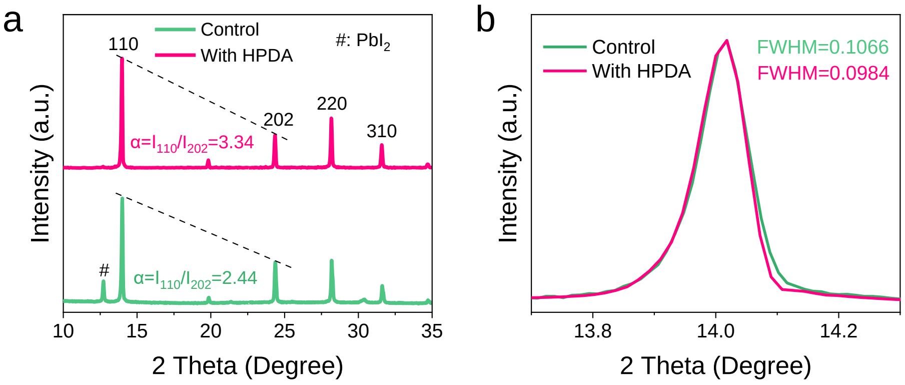

Supplementary Fig. 12. Crystal structure of the control and HPDA–modified perovskite films. (a) XRD patterns of perovskite films; (b) the full width at half maximum (FWHM) of the (110) peak.

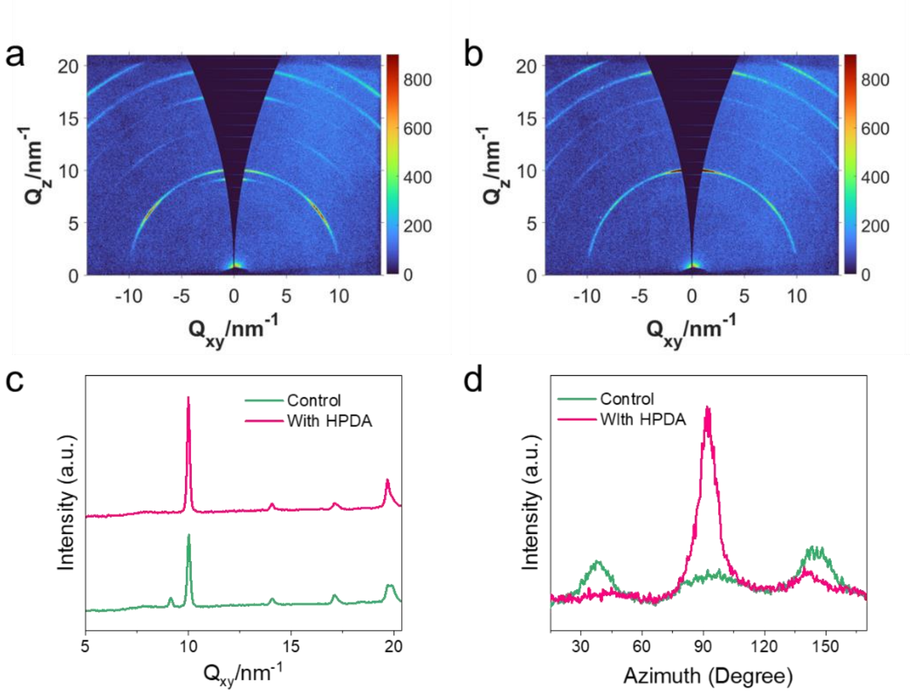

Supplementary Fig 13. GIWAXS characterization of perovskite films. (a) control and (b) HPDA-modified perovskite films. (c) GIWAXS q-integrated intensity curves for control and HPDA-modified perovskite films. (d) Azimuthal integrated curves of the (110) plane of the perovskite films without and with HPDA.

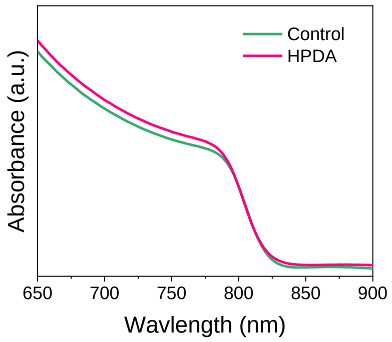

Supplementary Fig. 14. UV–Vis absorption spectra of the perovskite films with and without HPDA.

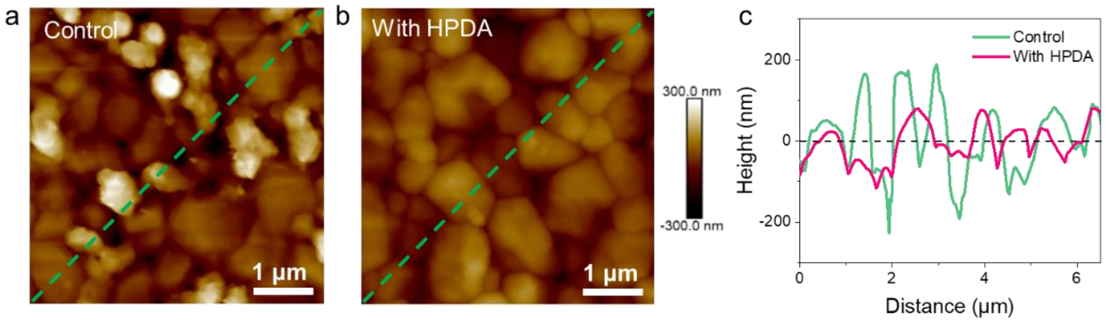

Supplementary Fig. 15. Surface roughness of the perovskite films. AFM images of (a) control and (b) HPDA-modified perovskite films; (c) The height curves vary with distance are recorded from the green solid line in the corresponding atomic force microscope (AFM) image.

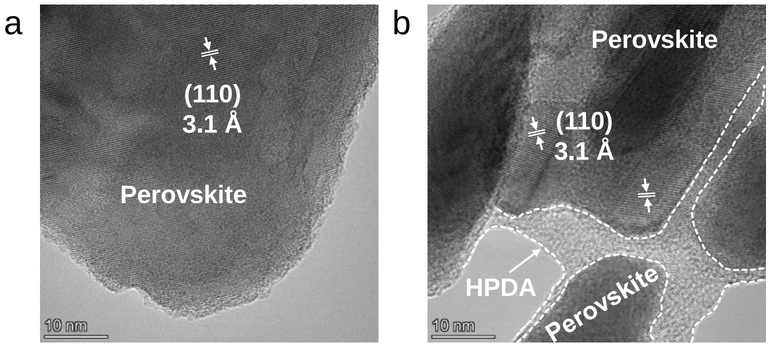

Supplementary Fig. 16.TEM images of the grain in the perovskite films. (a) without and (b) with HPDA.

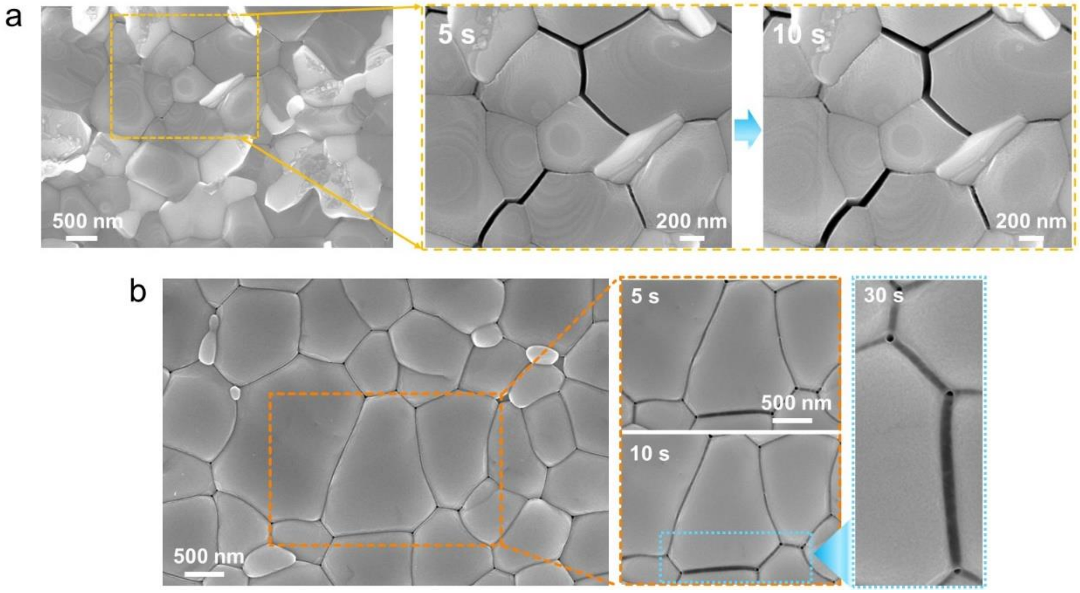

Supplementary Fig. 17. Time-dependent high-resolution SEM images of the perovskite grain boundaries under intense electron beam irradiation. (a) control and (b) HPDA-modified films.

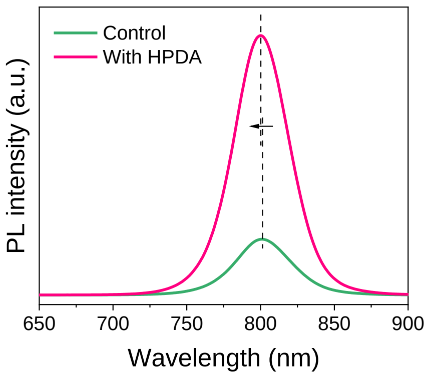

Supplementary Fig. 18. Steady-state PL spectra of the perovskite films with and without HPDA.

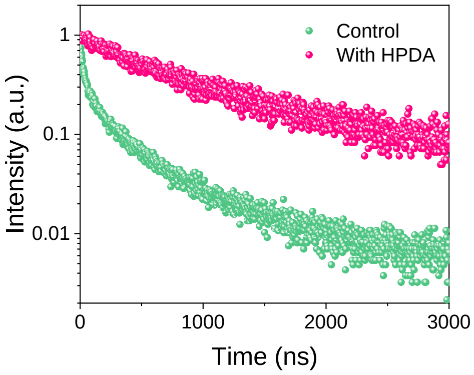

Supplementary Fig. 19. TRPL spectra of the perovskite films with and without HPDA.

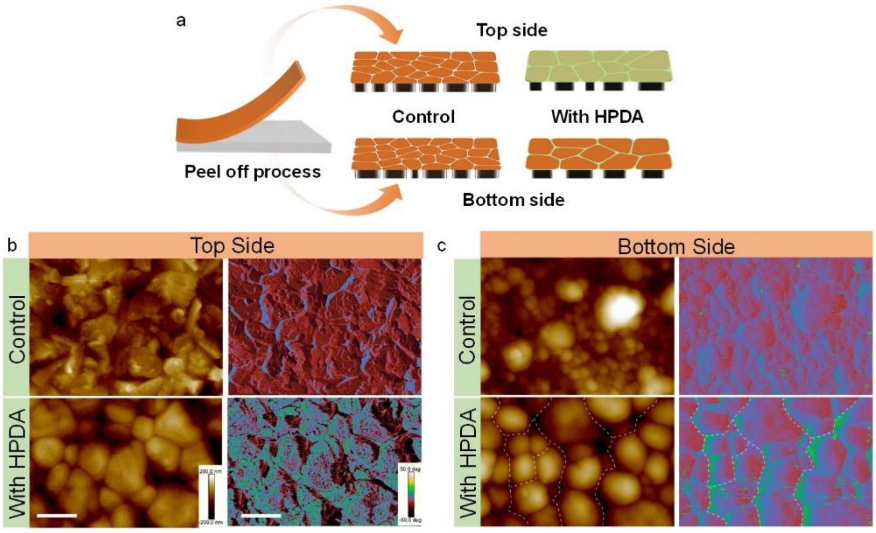

Supplementary Fig. 20. Characterization of the HPDA distribution in the perovskite films. (a) Schematic illustration of the film lift-off process for AFM characterizations of the top and bottom surfaces. AFM topography and corresponding phase images of top and bottom surfaces for (b) control and (c) HPDA-modified perovskite films. (Scale bar=1 μm)

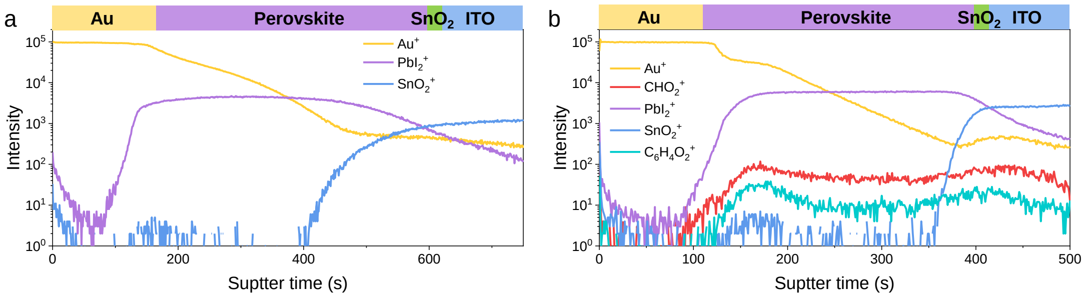

Supplementary Fig. 21. Time of flight secondary ion mass spectrometry (Tof-SMIS) depth profiles of the PSCs. (a) control and (b) HPDA-modified PSCs.

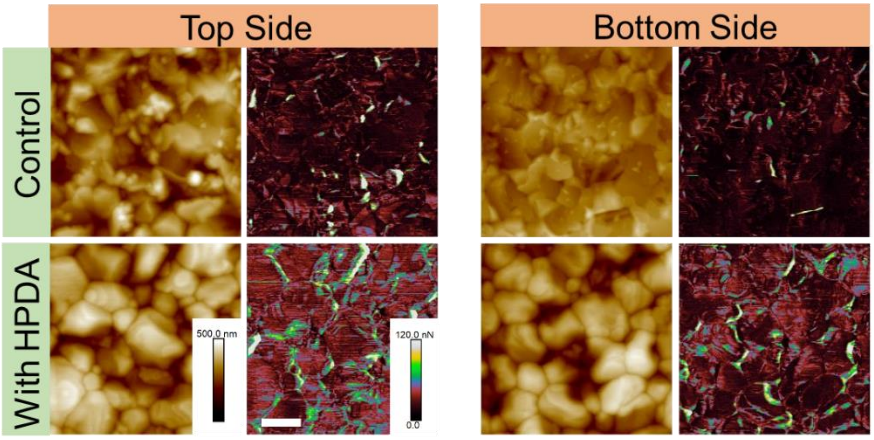

Supplementary Fig. 22. Adhesive force distribution characterization of the perovskite films. AFM morphology and corresponding AFM force images of top and bottom surfaces. (scale bar=1 μm)

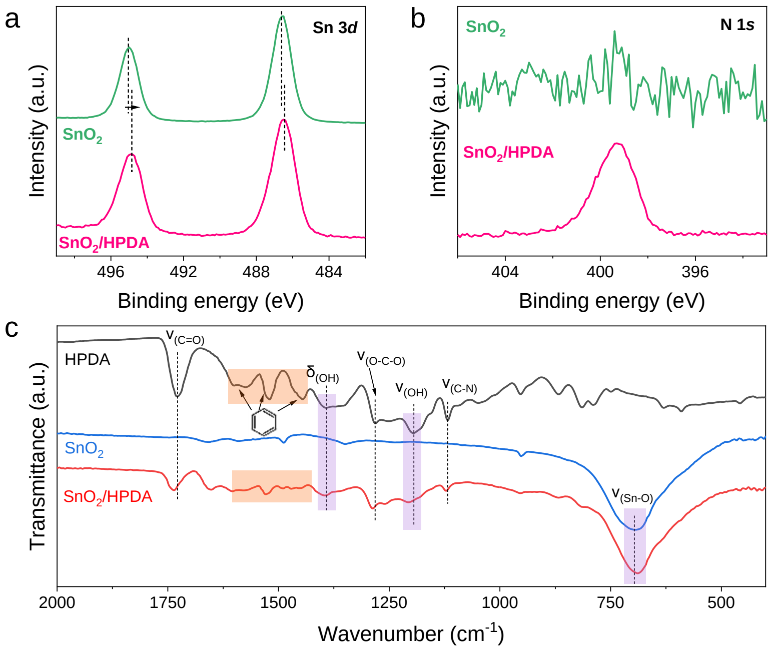

Supplementary Fig. 23. The interaction between HPDA and the SnO_2 layer. XPS of (a) Sn 3*d* and (b) N 1*s* peaks of SnO_2 and HPDA-modified SnO_2 films. (c) FTIR spectra of control and HPDA-modified SnO_2 films.

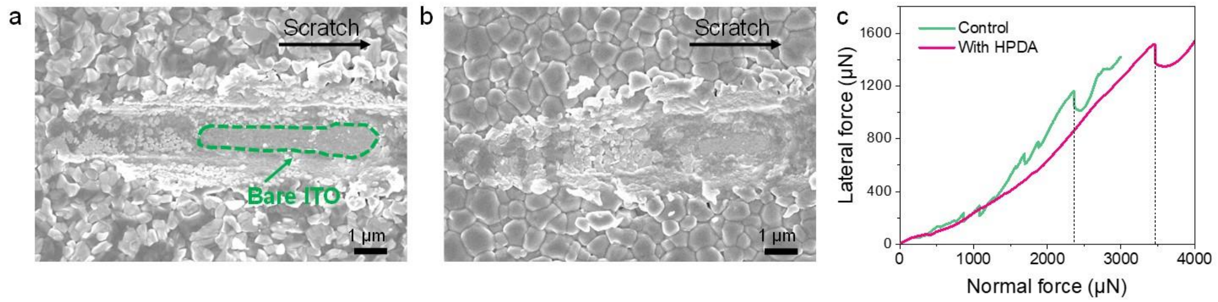

Supplementary Fig. 24. Interfacial bonding strength of the perovskite film to the SnO_2 ETL. SEM images of (a) control and (b) HPDA-modified perovskite films after nano scratch for adhesive strength test. (c) Scratch curve of perovskite film.

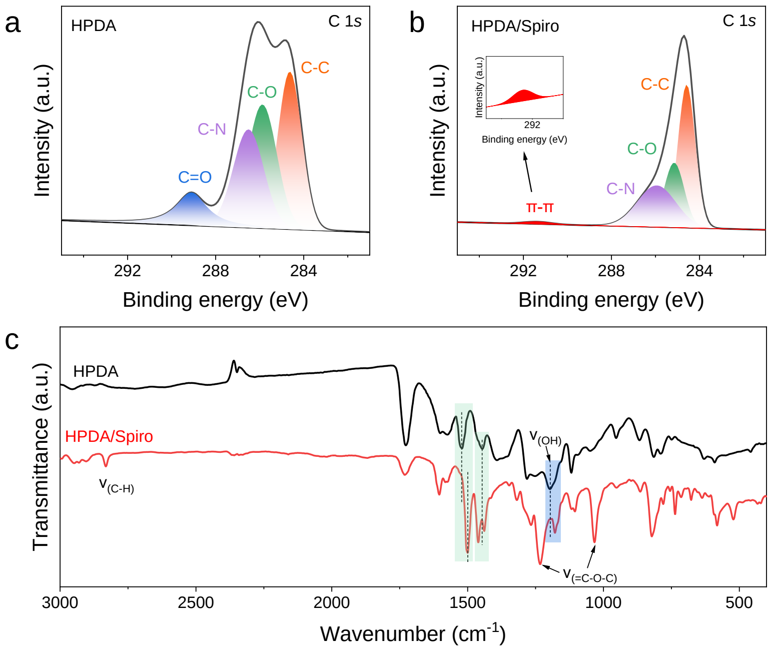

Supplementary Fig. 25. The interaction between HPDA and the Spiro-OMeTAD layer. XPS of C 1*s* peaks of (a) HPDA and (b) HPDA-doped with Spiro-OMeTAD. (c) FTIR spectra of HPDA and HPDA-doped with Spiro-OMeTAD.

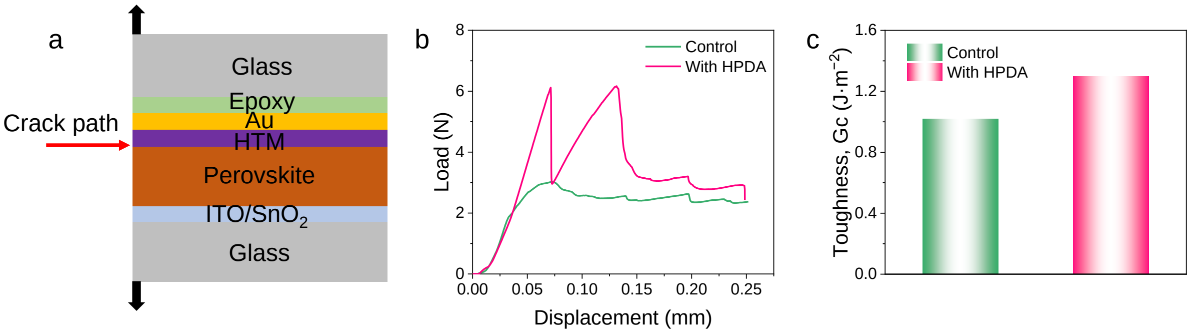

Supplementary Fig. 26. Mechanical robustness of perovskite/Spiro-OMeTAD interface. (a) Schematic illustration of double cantilever beam (DCB) test. (b) Representative P-△ curves for the measurement of G_C of the “sandwich” DCB specimens with and without HPDA modification. (c) Fracture energy of the PVK/HTL interface with and without HPDA modification.

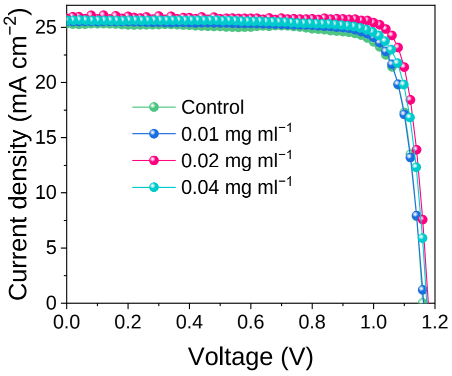

Supplementary Fig. 27. Photovoltaic performance of the PSCs with different concentration

(mg ml^{-1}) of HPDA.

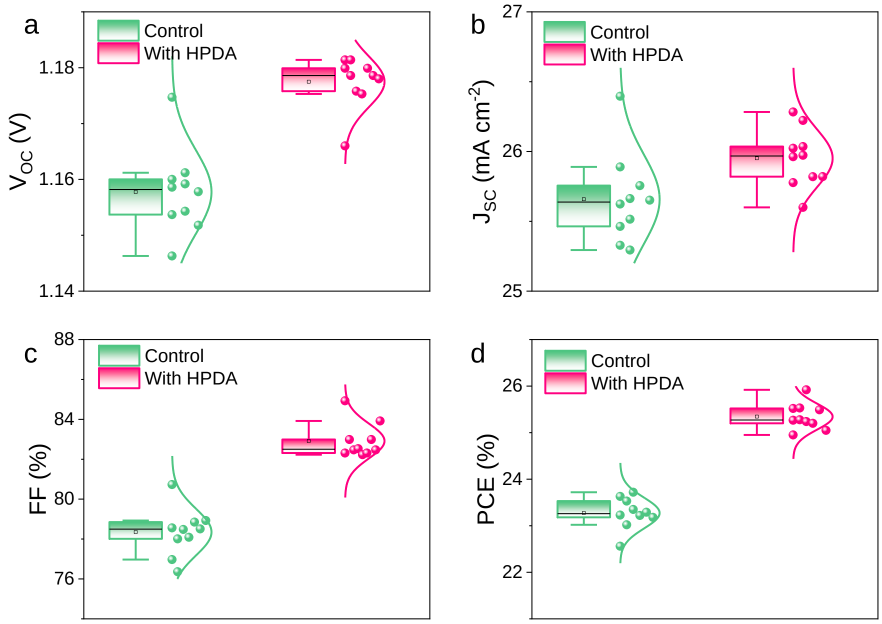

Supplementary Fig. 28. Photovoltaic parameter statistics of PSCs for the control and HPDA–modified devices with optimal HPDA concentration. (a) V_{OC}; (b) J_{SC}; (c) FF; (d) PCE. Error bars represent the standard deviations from the statistic results of two devices.

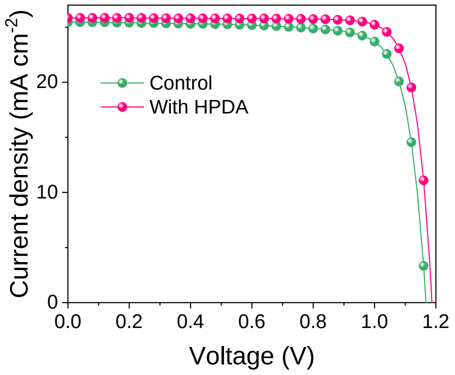

Supplementary Fig. 29. ***J–V* curves of the champion inverted p-i-n devices with and without HPDA.**

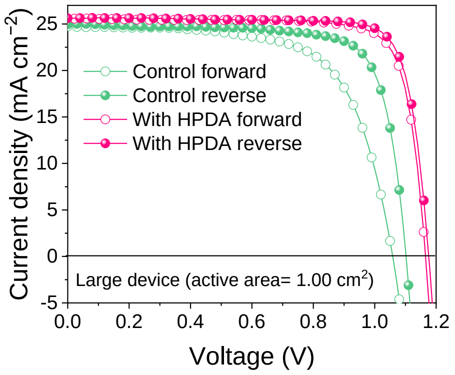

Supplementary Fig. 30. *J–V* curves of the champion control and HPDA-modified PSCs with large area of 1 cm^2.

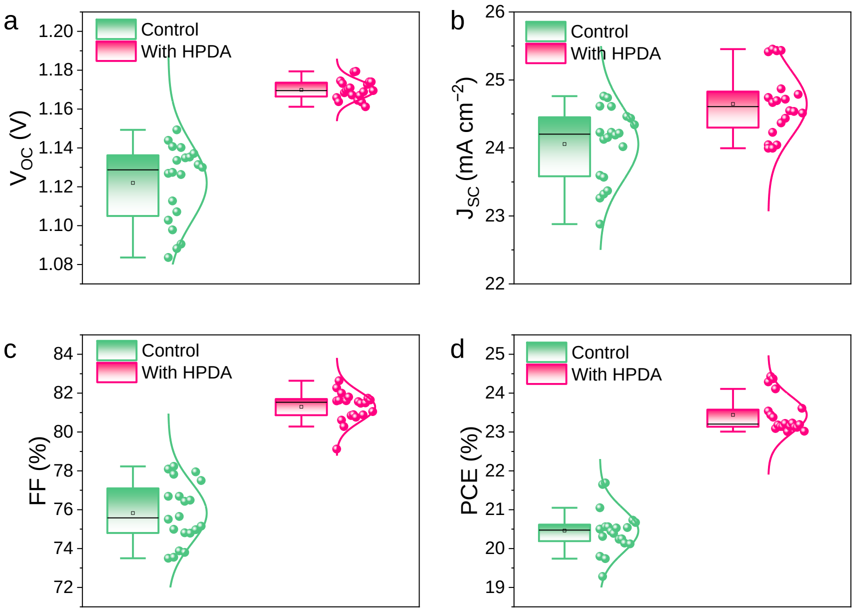

Supplementary Fig. 31. Photovoltaic parameter statistics of FPSCs for the control and HPDA–modified devices. (a) V_{OC}; (b) J_{SC}; (c) FF; (d) PCE. Error bars represent the standard deviations from the statistic results of two devices.

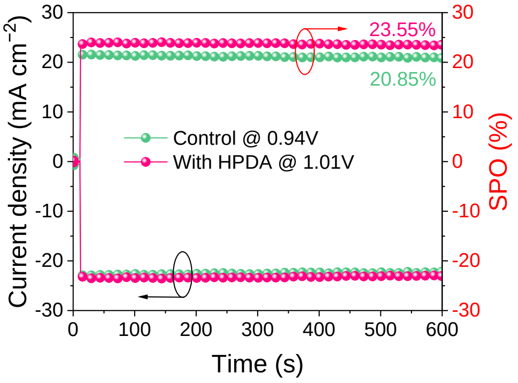

Supplementary Fig. 32. Stabilized photocurrent and power output of the FPSCs with and without HPDA modification.

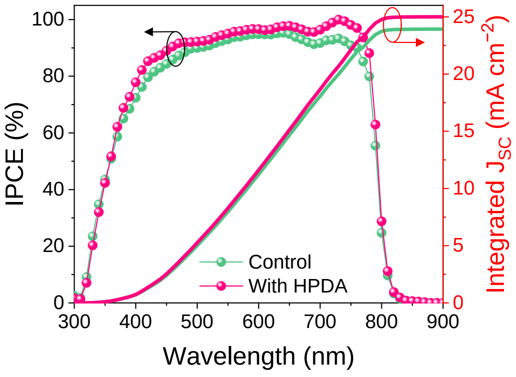

Supplementary Fig. 33. External quantum efficiency (EQE) spectra and integrated current of the FPSCs with and without HPDA modification.

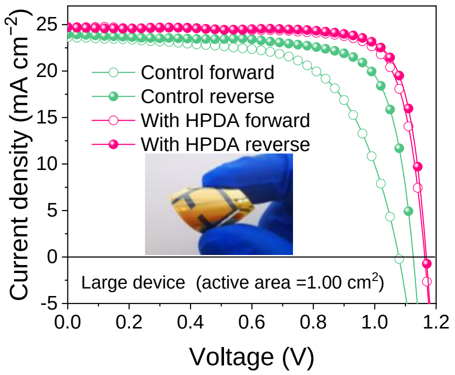

Supplementary Fig. 34. ***J–V* curve of the champion control and HPDA-modified FPSCs with large area of 1 cm^2.** Inset shows a photo of the large area solar cell.

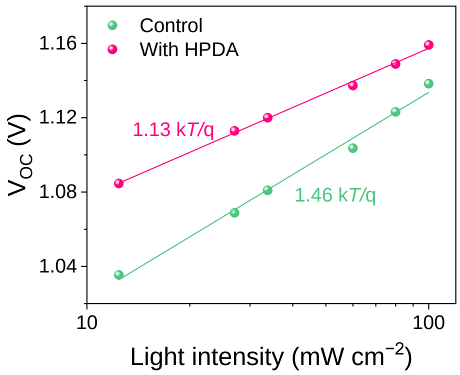

Supplementary Fig. 35. Suns-V_{OC} characterizations of the control and HPDA-modified PSCs.

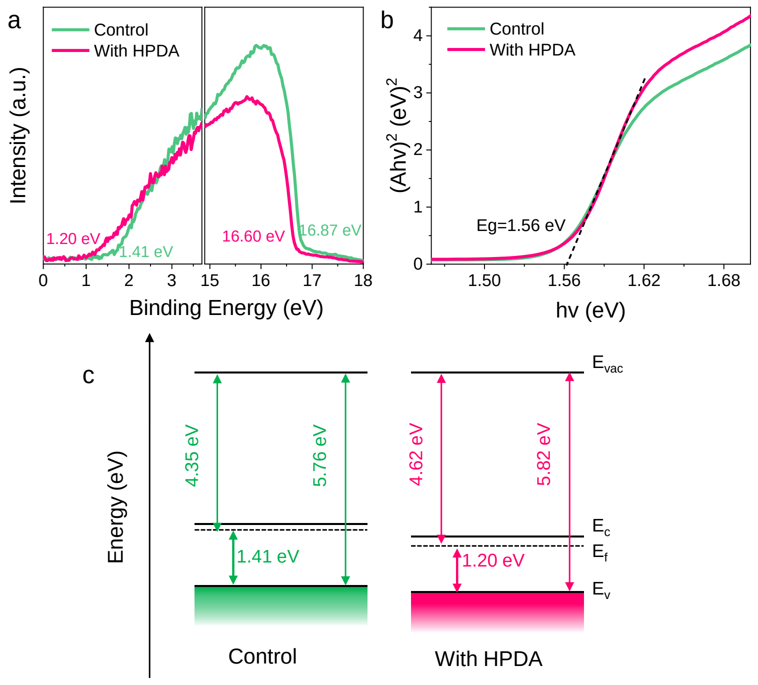

Supplementary Fig. 36. Energy levels of the perovskite films with and without HPDA

modification. (a) UPS data of the control and HPDA-modified perovskite films; (b) Tauc plots of the control and HPDA-modified perovskite films; (c) Energy band diagram.

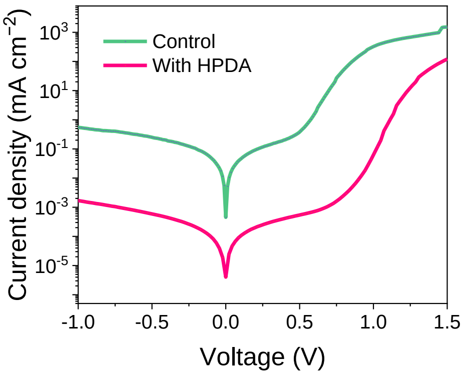

Supplementary Fig. 37. Dark *J–V* curves of the PSCs with and without HPDA modification.

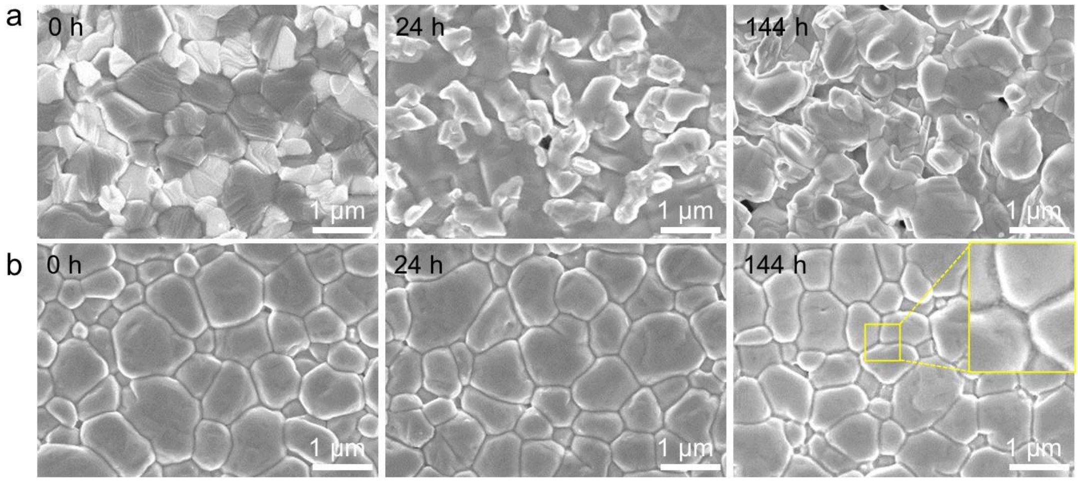

Supplementary Fig. 38. SEM images of the perovskite films after exposed to 85% relative humidity for different time. (a) control and (b) HPDA-modified films.

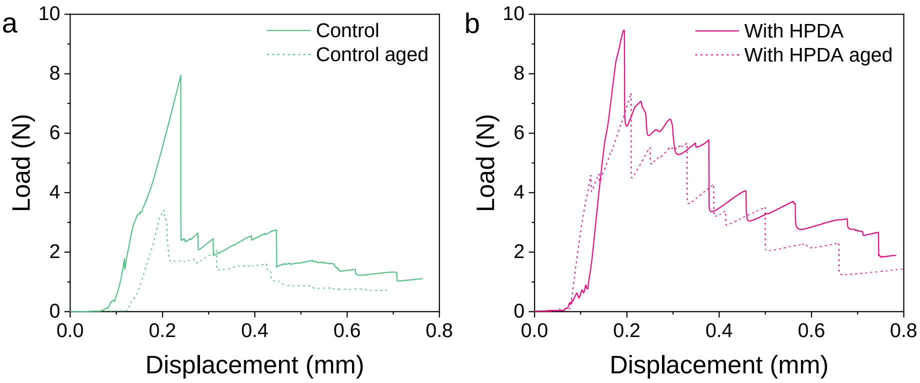

Supplementary Fig. 39. Mechanical robustness of the perovskite films. Representative P-△ curves for the measurement of G_C of the “sandwich” DCB specimens of (a) the control and (b) films with HPDA modification.

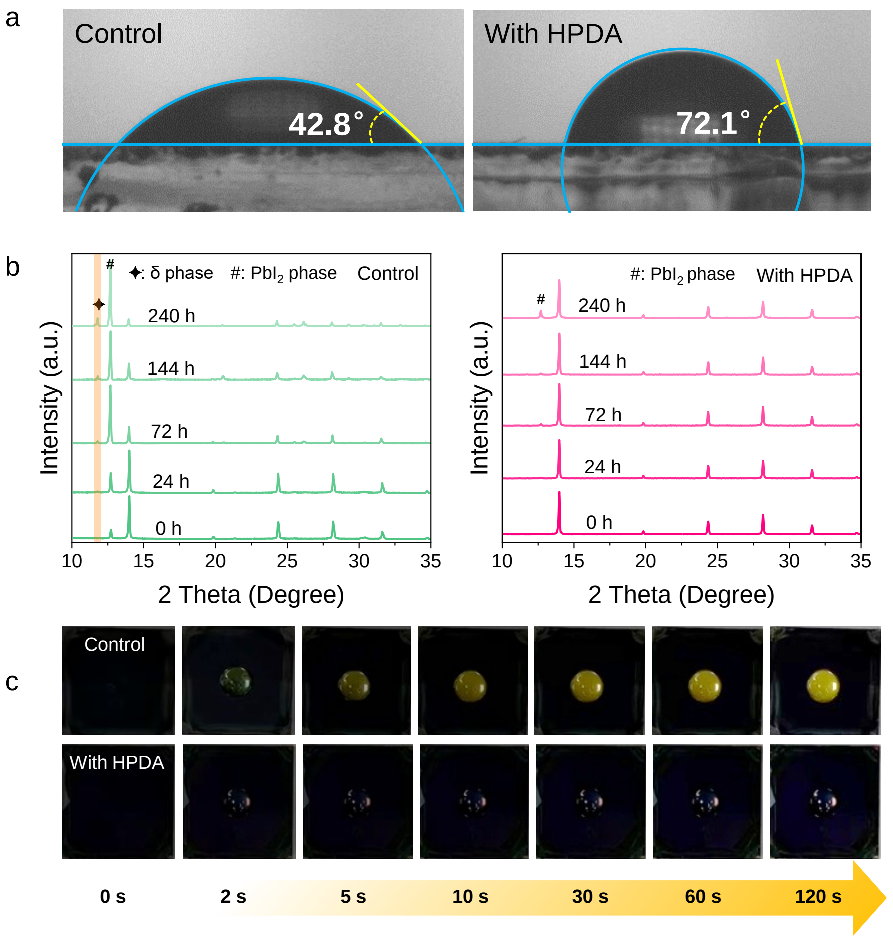

Supplementary Fig. 40. Humidity stability of the perovskite films. (a) Contact angle measurements of the perovskite films with and without HPDA modification showing excellent hydrophobic properties of HPDA with increased contact angle; (b) XRD of the control and HPDA-modified perovskite films as a function of time under relative humidity of 85%, exhibiting more distinct diffraction peak of lead iodide and δ-$FAPbI_3$ signal in the control perovskite films compared to the HPDA-modified films; (c) Photographs of the control and HPDA-modified perovskite films with water drops.

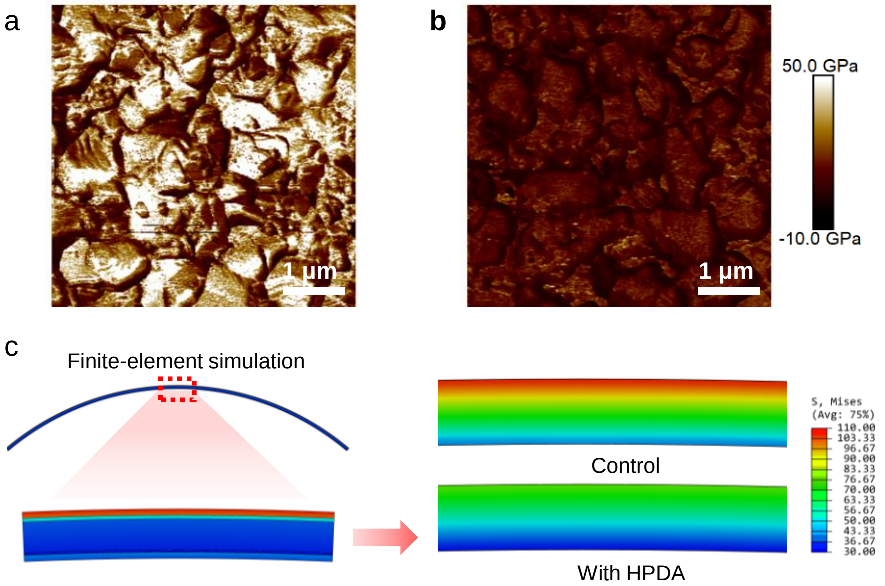

Supplementary Fig. 41. Calculation of the stress in the perovskite films during bending. Young's modulus of the perovskite layers characterized using AFM (a) without and (b) with HPDA modification. (c) Finite-element simulation of the devices with and without HPDA modification, showing that HPDA layer can significantly reduce the stress in the perovskite layers and improve the mechanical durability of the devices.

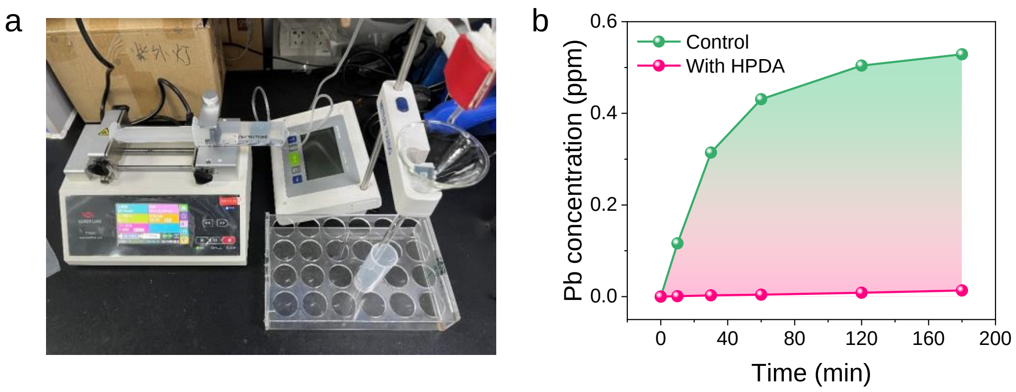

Supplementary Fig. 42. Lead leakage measurements. (a) Photograph of homemade equipment to study the lead leakage from damaged PSCs under rainfall conditions. (b) Pb concentration in the contaminated water measured by ICP-MS.

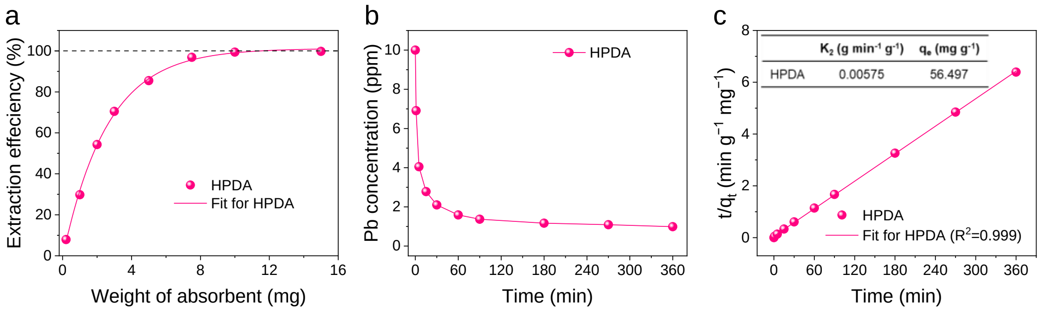

Supplementary Fig. 43. Pb^{2+} absorption properties of HPDA. (a) Influence of absorbent weight

toward Pb^{2+} extraction efficiency. (b) Pb^{2+} sorption kinetics and the corresponding absorbed Pb^{2+} amount of HPDA films. (c) The kinetics fitting curves of HPDA films from a pseudo-second-order mode.

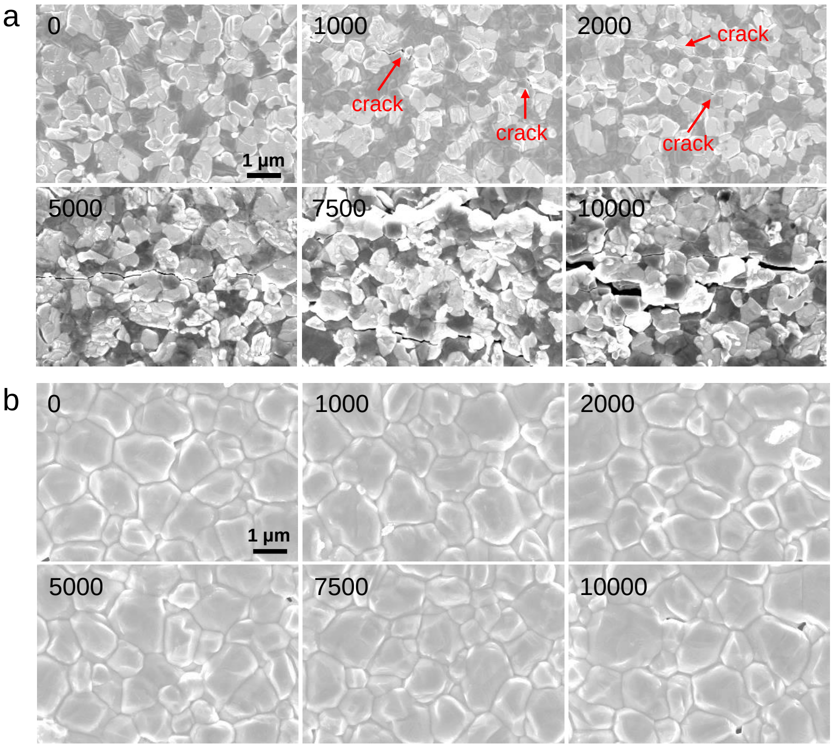

Supplementary Fig. 44. Mechanical robustness of the perovskite films. SEM images of (a) control and (b) HPDA-modified perovskite film after bending for different cycles.

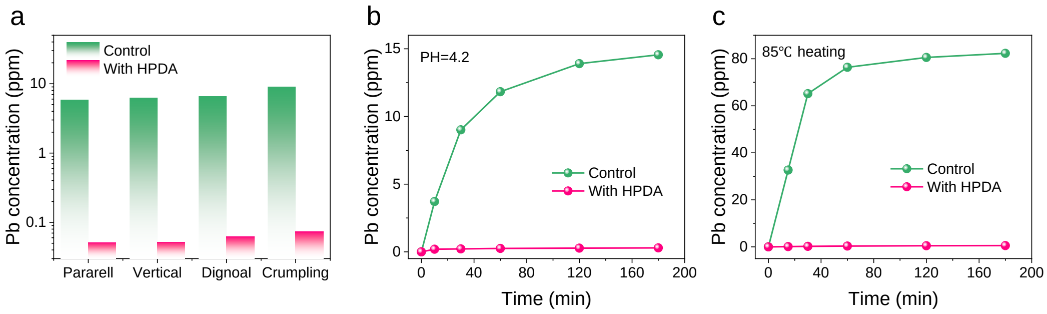

Supplementary Fig. 45. **Lead leakage behaviors of the perovskite films.** (a) Pb-leakage behavior of the perovskite films after bending with different directions (parallel, vertical and diagonal to the electrode direction) for 500 cycles or crumpling for 200 times and then water dripped for 30 min, respectively. (b) Acidic water dripping test of the control and HPDA-modified perovskite films. (c) Further heat water soaking test at 85 °C of the control and HPDA-modified films after acidic rainfall test.

Supplementary Table 1. The fitting parameters of the TRPL spectra.

Samples	**A_1 (%)**	**τ_1 (ns)**	**A_2 (%)**	**τ_2 (ns)**	**τ_{AVE} (ns)**
Control	22.92	117.6	77.08	1537.9	408.0
With HPDA	44.55	473.4	55.45	1462.4	489.9

Supplementary Table 2 Surface adhesion force of perovskite film with and without HPDA modification.

Samples	Top side	Bottom side
Control	23.8 nN	16.7 nN
With HPDA	43.0 nN	33.0 nN

Supplementary Table 3. Statistical photovoltaic parameters of the devices with different concentration of HPDA modification.

Concentration (mg ml^{-1})	V_{OC} (V)	J_{SC} (mA cm^{-2})	*FF*	PCE (%)
0	1.160	25.33	80.73	23.72
0.01	1.163	25.55	81.08	24.10
0.02	1.178	25.82	84.93	25.92
0.04	1.173	25.63	82.05	24.68

Supplementary Table 4. Statistical photovoltaic parameters of the devices with and without HPDA modification.

Samples	V_{OC} (V)	J_{SC} (mA cm^{-2})	*FF*	PCE (%)
Control	1.159 ± 0.013	24.20 ± 0.42	76.16 ± 1.92	20.81 ± 0.64
With HPDA	1.177 ± 0.004	25.95 ± 0.20	82.91 ± 0.87	25.34 ± 0.27

Supplementary Table 5. Champion photovoltaic parameters of the flexible devices with and without HPDA modification.

Samples	V_{OC} (V)	J_{SC} (mA cm^{-2})	*FF*	PCE (%)
Control-F	1.086	24.57	73.39	19.6
Control-R	1.137	24.61	77.50	21.69
With HPDA-F	1.172	25.41	81.50	24.29
With HPDA-R	1.174	25.45	81.74	24.43

Supplementary Table 6. Statistical photovoltaic parameters of the flexible devices with and without HPDA modification.

Samples	V_{OC} (V)	J_{SC} (mA cm^{-2})	*FF*	PCE (%)
Control	1.122 ± 0.020	24.05 ± 0.54	75.82 ± 1.57	20.46 ± 0.57
With HPDA	1.169 ± 0.004	24.64 ± 0.48	81.29 ± 0.77	23.44 ± 0.47

Supplementary Table 7. Energy levels information of control and HPDA-modified perovskite film.

Samples	E_g	E_l	E_{cutoff}	W_F	E_{Fermi}	E_{VB}	E_{CB}
Control	1.56	1.41	16.87	4.35	-4.35	-5.76	-4.20
With HPDA	1.56	1.20	16.60	4.62	-4.62	-5.82	-4.26

Supplementary Note 1

GIXRD Residual strain gradient measurement

X-ray diffraction (XRD) represents a common technique to evaluate residual stress/strain gradients along the surface normal direction in polycrystalline thin films and coatings from measured X-ray elastic strains using the $\sin^2\psi$ technique. The measured lattice spacing d and X-ray elastic strains ε represent volume-average quantities which depend on the actual stress/strain depth profile, X-ray penetration depth, reflection plane (hkl) and experiment geometry. In general, d and ε can be related by $\varepsilon=(d-d_0)/d_0$. According to the Braggs law, Hooke's law and equations of equilibrium, the classic sin2ψ equation about stress σ and 2θ can be obtained as follow:

$$\sigma = -\frac{E}{2(1+\nu)}\frac{\pi}{180}\cot\theta_0\frac{\partial(2\theta)}{\partial\sin^2\psi} \qquad (1)$$

where E and υ are Young's modulus and Poisson's ratio of the thin film, respectively. θ_0 is the diffraction peak for stress free perovskite (hkl) crystal plane and θ is the diffraction peak for the actual perovskite thin films. The ψ is the angle the diffraction vector with respect to the sample normal direction.

Here we define

$$C = -\frac{E}{2(1+\nu)}\frac{\pi}{180}\cot\theta_0 \qquad k' = \frac{\partial(2\theta)}{\partial\sin^2\psi}$$

We can use C and k' to simplify the formula. When determining the diffraction angle, C is a constant and there is a linear function between 2θ and $\sin^2\psi$, k' is the slope. Then, we can transfer the formula (1) to

$$\varepsilon = \frac{\sigma}{E} = \frac{C}{E}k' \quad (2)$$

According to the formula (2), the state of the residual stress σ and macroscopic residual strain ε can be judged by the slope of 2θ-$\sin^2\psi$ line. When $k' < 0$, the ε value is positive and it is tensile strain/stress, $k' > 0$, the ε value is negative then it is compressive strain/stress, the magnitude of strain/stress is determined by the value of the slope.

By fitting the d-spacing as a function of ψ, we calculate film stress as:

$$\frac{\partial d_\psi}{\partial\sin^2\psi} = \frac{1+\nu}{E}\sigma\cdot d_n$$

where σ is the bulk film stress, d_n is the d-spacing along the surface normal, i.e. for ψ=0

Using this method, we find the following values for film stress.

Supplementary Note 2

Mechanical Testing

DCB specimens were loaded under displacement control in a universal testing machine (CMT 5305) from which a load, P, versus displacement, Δ, curve was recorded. The adhesive fracture energy, Gc (J m^{-2}), was measured in terms of the critical value of the applied strain energy release rate, G. Gc can be expressed in terms of the critical load, Pc, at which crack growth occurs, the crack length, a, the plane-strain elastic modulus, E, of the substrates and the specimen dimensions: width, B and half-thickness, h. Gc was calculated from Equation: $G_c = \frac{12Pc^2a^2}{B^2Eh^3}\left(1 + 0.64\frac{h}{a}\right)^2$. An estimate of the crack length was experimentally determined from a measurement of the elastic compliance, dΔ/dP, using the compliance relationship in Equation: $a = \left(\frac{d\Delta}{dP} * \frac{BEh^3}{8}\right)^{\frac{1}{3}} - 0.64h$. All Gc testing was carried out in laboratory air environment at ≈25 °C and ≈45% R.H. The specimen was loaded in tension with a displacement rate of 1 mm min^{-1} until reaching Pc to calculate dΔ/dP.
