# Supplementary material for: Boosting mechanical durability under high humidity by bioinspired multisite polymer for high-efficiency flexible perovskite solar cells
Source: Nat Commun. 2025 Feb 19;16:1771. doi: 10.1038/s41467-025-57102-3 (PMC11840045; doi:10.1038/s41467-025-57102-3)
Supplement: Supplementary file 2 — Reporting Summary [file 41467_2025_57102_MOESM2_ESM.pdf]

nature portfolio

Corresponding author(s): Zhen Li

# Solar Cells Reporting Summary

Nature Portfolio wishes to improve the reproducibility of the work that we publish. This form is intended for publication with all accepted papers reporting the characterization of photovoltaic devices and provides structure for consistency and transparency in reporting. Some list items might not apply to an individual manuscript, but all fields must be completed for clarity.

For further information on Nature Research policies, including our data availability policy, see Authors & Referees.

## • Experimental design

**Please check the following details are reported in the manuscript, and provide a brief description or explanation where applicable.**

### 1. Dimensions

| Item | Yes/No | Description / Explanation |
| --- | --- | --- |
| Area of the tested solar cells | ☒ Yes ☐ No | The size of device is defined by metal masks. / *Explain why this information is not reported/not relevant.* |
| Method used to determine the device area | ☒ Yes ☐ No | All J-V dates were tested using metal masks. / *Explain why this information is not reported/not relevant.* |

### 2. Current-voltage characterization

| Item | Yes/No | Description / Explanation |
| --- | --- | --- |
| Current density-voltage (J-V) plots in both forward and backward direction | ☒ Yes ☐ No | Figure 3a for rigid device. Fig. 3d for flexible device. |
| Voltage scan conditions | ☒ Yes ☐ No | 0.1 V/s, mentioned in method section. / *Explain why this information is not reported/not relevant.* |
| Test environment | ☒ Yes ☐ No | in N2 glove box at room temperature / *Explain why this information is not reported/not relevant.* |
| Protocol for preconditioning of the device before its characterization | ☐ Yes ☒ No | *Provide a description of the protocol.* / No precondition is applied. |
| Stability of the J-V characteristic | ☒ Yes ☐ No | Long term stability test was carried out in a N2 glovebox under 100 mW/cm2 illumination from white LED, using a solar cell stability testing system (PR-SCCS-C8Q, Puri Materials). / *Explain why this information is not reported/not relevant.* |

### 3. Hysteresis or any other unusual behaviour

| Item | Yes/No | Description / Explanation |
| --- | --- | --- |
| Description of the unusual behaviour observed during the characterization | ☒ Yes ☐ No | J-V hysteresis was observed and reported / *Explain why this information is not reported/not relevant.* |
| Related experimental data | ☒ Yes ☐ No | J-V hysteresis was observed and reported / *Explain why this information is not reported/not relevant.* |

### 4. Efficiency

| Item | Yes/No | Description / Explanation |
| --- | --- | --- |
| External quantum efficiency (EQE) or incident photons to current efficiency (IPCE) | ☒ Yes ☐ No | The incident photocurrent conversion efficiency (IPCE) spectra were measured using a quantum efficiency testing system (QE-R 3011, Enli Tech). / *Explain why this information is not reported/not relevant.* |
| A comparison between the integrated response under the standard reference spectrum and the response measure under the simulator | ☒ Yes ☐ No | The integrated current density match well with the J-V curves (<3 % deviation) / *Explain why this information is not reported/not relevant.* |

For tandem solar cells, the bias illumination and bias voltage used for each subcell

- [ ] Yes — *Provide a description of the measurement conditions.*
- [x] No — *Explain why this information is not reported/not relevant.*

## 5. Calibration

Light source and reference cell or sensor used for the characterization

- [x] Yes — under AM 1.5G, 100 mW/cm2 illumination generated by a solar simulator (Sol3A 94063A, Newport)
- [ ] No — *Explain why this information is not reported/not relevant.*

Confirmation that the reference cell was calibrated and certified

- [x] Yes — The solar simulator was calibrated using a KG-5 filtered standard solar cell (SRC-2020, NREL calibrated)
- [ ] No — *Explain why this information is not reported/not relevant.*

Calculation of spectral mismatch between the reference cell and the devices under test

- [x] Yes — The standard Si solar cell was filtered by a KG-5 filter to simulate spectral response of the PSCs.
- [ ] No — *Explain why this information is not reported/not relevant.*

## 6. Mask/aperture

Size of the mask/aperture used during testing

- [x] Yes — 0.045 cm2 (3*15 mm) for small area device, and 1 cm2 (8*12.5 mm) for large area device
- [ ] No — *Explain why this information is not reported/not relevant.*

Variation of the measured short-circuit current density with the mask/aperture area

- [x] Yes — 25.92 for 0.045 cm2 rigid device and 24.68 for 1 cm2 rigid device. 24.43 for 0.045 cm2 flexible device and 23.01 for 1 cm2 flexible device.
- [ ] No — *Explain why this information is not reported/not relevant.*

## 7. Performance certification

Identity of the independent certification laboratory that confirmed the photovoltaic performance

- [ ] Yes — *Identify the independent certification laboratory.*
- [x] No — The device was not independently certified.

A copy of any certificate(s)

- [ ] Yes — *Certificate copies should be provided in the Supplementary information. Please state the supplementary item number.*
- [x] No — The device was not independently certified.

## 8. Statistics

Number of solar cells tested

- [x] Yes — At least 10 devices were tested to generate statistical results
- [ ] No — *Explain why this information is not reported/not relevant.*

Statistical analysis of the device performance

- [x] Yes — Figure S23 for rigid devices and Figure S25 for flexible devices.
- [ ] No — *Explain why this information is not reported/not relevant.*

## 9. Long-term stability analysis

Type of analysis, bias conditions and environmental conditions

- [x] Yes — Long term stability test was carried out in a N2 glovebox under 100 mW/cm2 illumination from white LED. The maximum power points of the PSCs were tracked using a solar cell stability testing system (PR-SCCS-C8Q, Puri Materials).
- [ ] No — *Explain why this information is not reported/not relevant.*
